# Signal Enhancement of Low Magnetic Field Magnetic Resonance Image Using a Conventional- and Cyclic-Generative Adversarial Network Models With Unpaired Image Sets

**DOI:** 10.3389/fonc.2021.660284

**Published:** 2021-05-11

**Authors:** Denis Yoo, Yuni Annette Choi, C. J. Rah, Eric Lee, Jing Cai, Byung Jun Min, Eun Ho Kim

**Affiliations:** ^1^ Artificial Intelligence Research Lab, Talos, Sheung Wan, Hong Kong; ^2^ Department of Health Technology & Informatics, Hong Kong Polytechnic University, Hung Hom, Hong Kong; ^3^ Department of Radiation Oncology, Chungbuk National University Hospital, Cheongju, South Korea; ^4^ Department of Biochemistry, School of Medicine, Daegu Catholic University, Daegu, South Korea

**Keywords:** conventional-GAN, cyclic-GAN, enhancement of MR image, low magnetic field, Magnetic Resonance Image (MRI)

## Abstract

In this study, the signal enhancement ratio of low-field magnetic resonance (MR) images was investigated using a deep learning-based algorithm. Unpaired image sets (0.06 **Tesla** and 1.5 **Tesla** MR images for different patients) were used in this study following three steps **workflow**. In the first step, the deformable registration of a 1.5 **Tesla** MR image into a 0.06 **Tesla** MR image was performed to ensure that the shapes of the unpaired set matched. In the second step, a cyclic-generative adversarial network (GAN) was used to generate a synthetic MR image of the original 0.06 **Tesla** MR image based on the deformed or original 1.5 **Tesla** MR image. Finally, an enhanced 0.06 **Tesla** MR image could be generated using the conventional-GAN with the deformed or synthetic MR image. The results from the optimized flow and enhanced MR images showed significant signal enhancement of the anatomical view, especially in the nasal septum, inferior nasal choncha, nasopharyngeal fossa, and eye lens. The signal enhancement ratio, signal-to-noise ratio (SNR) and correlation factor between the original and enhanced MR images were analyzed **for the evaluation of the image quality**. A combined method using conventional- and cyclic-GANs is a promising approach for generating enhanced MR images from low-magnetic-field MR.

## Introduction

Magnetic resonance imaging (MRI)-based contouring is a standard practice in radiotherapy (1–4). Recently, the use of MRI has been extended to the entire external photon radiotherapy (RT) treatment planning workflow (5–16). In addition, for proton therapy, the feasibility of magnetic resonance (MR)-only treatment planning has been investigated (17, 18). In this MR-only RT workflow, the development of techniques for determining the electron density in MRI-derived substitute-computed tomography (sCT) images have been investigated, including atlas-based and deep learning-based methods (18–22). For synthetic CT generation, Han’s generative adversarial network (GAN) model (20) or modified models (18, 21, 22) have generally been used with sets of two-paired images (an MR/CT image set for the same patient taken within one day). In this conventional GAN, the CT images are the ground truth, and the MR images are the input images.

In addition to sCT, the generation of synthetic MRI for image-to-image translation of T1- and T2-weighted MR images (23) has been investigated using a cyclic-GAN algorithm. The neural network training is commonly supervised, that is, the training requires the corresponding ground truth for each input sample. In image-to-image translation, this implies that paired images from both the source and target domains are needed. To alleviate this constraint, the cyclic-GAN (24) and UNIT (25) can work with unpaired training data. In this cyclic-GAN, two input images tend to resemble each other.

1.5 **Tesla** has become the standard clinical machine even in very small hospitals, almost completely replacing the older lower field strength (0.2–1 **Tesla**) machines that played an important role in the development of MRI during the 1980s (26). Recent advances in MRI technology have allowed for data acquisition at low magnetic field strengths. MRI scanners operating at low field strengths allow for open geometry designs that can ease patient handling and positioning and are compatible with nearby ferromagnetic materials, enabling scanning outside of the controlled access environment of an MRI suite (27). Sheth et al., recently developed and deployed a novel bedside neuroimaging solution and reported the results (28). They used a 0.06 **Tesla** portable MR (hyperfine MR) machine for the assessment of brain injury. This portable MR machine was very efficient, especially for patients admitted to an intensive care unit. Though this technology is very promising, improvement of the image quality is necessary to apply this MRI to a more wide area; and to increase the image quality, a deep learning model can be a useful option.

To generate sCT from MR images, the image quality of the MRI must be assured, however, the current situation of the low magnetic field-based MR images were not of the quality required for the sCT generation. Thus, the quality of MRI from low-magnetic-field MR needs to be improved for the clinical usage of the MR-only RT workflow. Also, paired image sets for the training of the deep learning algorithm are not suitable in some clinical situations. However, the study of deep learning model-based training with unpaired image set has not been fully investigated yet. This study investigated the MR signal enhancement of 0.06 Tesla MRI using deep learning-based combined models (cyclic-GAN and conventional-GAN) with unpaired image sets. We suggested a clinical workflow using deformable registration and a deep learning-based combined model consisting of cyclic- and conventional-GAN. Also, three kinds of methods were compared to find an optimal workflow for the enhancement of the MR images.

## Method

### Patient Data and Matching Unpaired Image Set

**Figure 1:**
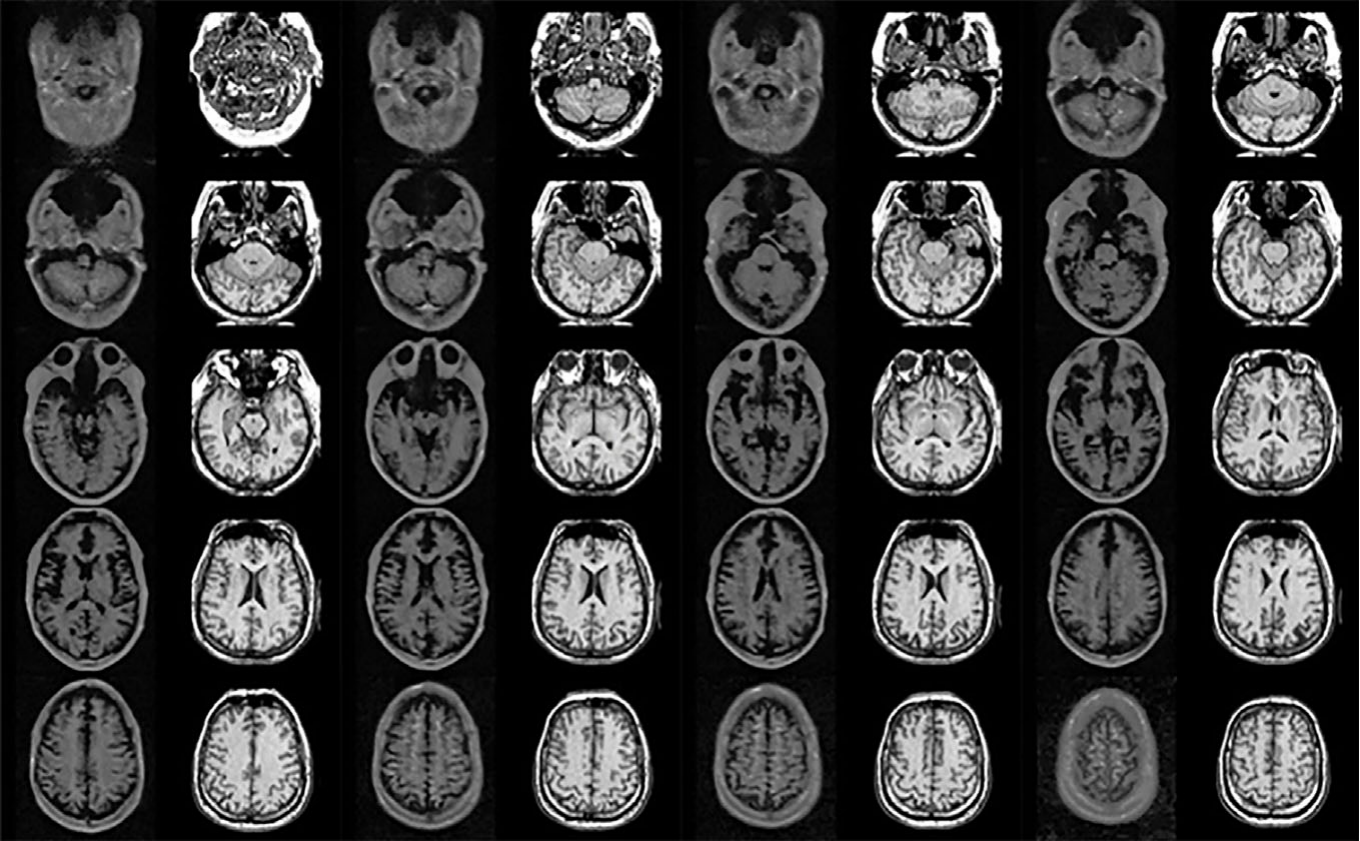
Unpaired image set used for training (left and right sides show 0.06 **Tesla** and 1.5 **Tesla** MR images, respectively).

This study used axial 0.06 Tesla MR images from the Hyperfine website (https://hyperfine.io/clinical with 26 images for T1-weighted (T1W) used for training and evaluation. In addition, 1.5 Tesla MR images from another website (http://www.med.harvard.edu/aanlib/home.htm) were used as reference images with the same number of slices. These unpaired images were matched to each other by a radiologist to ensure that they were as similar as possible. **Figure 1** shows an unpaired image set containing a 0.06 Tesla original MR image and 1.5 Tesla original MR image. For all the images, an image size of 256 × 256 and depth of 255 (8 bit) was used. The detailed imaging parameters for the 0.06 Tesla MRI are shown in (28). 0.06 Tesla MR images were acquired using an 8-channel head coil. The MRI used a biplanar, 3-axis gradient system with a peak amplitude of 26 mT/m (on the z-axis) and 25 mT/m (on the x-axis and y-axis). The scanning parameters were controlled using a computer interface (iPad Pro, third-generation; Apple). The following pulse 3-dimensional sequences were used: T1W fast spin echo (FSE) (repetition time [TR], 1500 milliseconds; time to echo [TE], 6 milliseconds; inversion time [TI], 300 milliseconds; 1.5 × 1.5 × 5-mm resolution; 36 slices). Examinations were acquired in the axial, sagittal, and coronal planes. In this study, the main aim is to study the enhancement of the brain structure in MRI, thus normal whole-brain images were used for both 0.06 and 1.5 Tesla MR images.

In hyperfine MRI (0.06 Tesla MRI), the overall intensity is low compared to 1.5 Tesla MRI, and the eye lens and nasal structure were not visible. The slices had different shapes in the 0.06 Tesla and 1.5 Tesla images, as well as different positions sizes. In this study, cyclic-GAN with and without deformable registration was applied to investigate the match of the unpaired image set. The detailed workflow and methods are presented in the next section.

### Workflow

**Figure 2:**
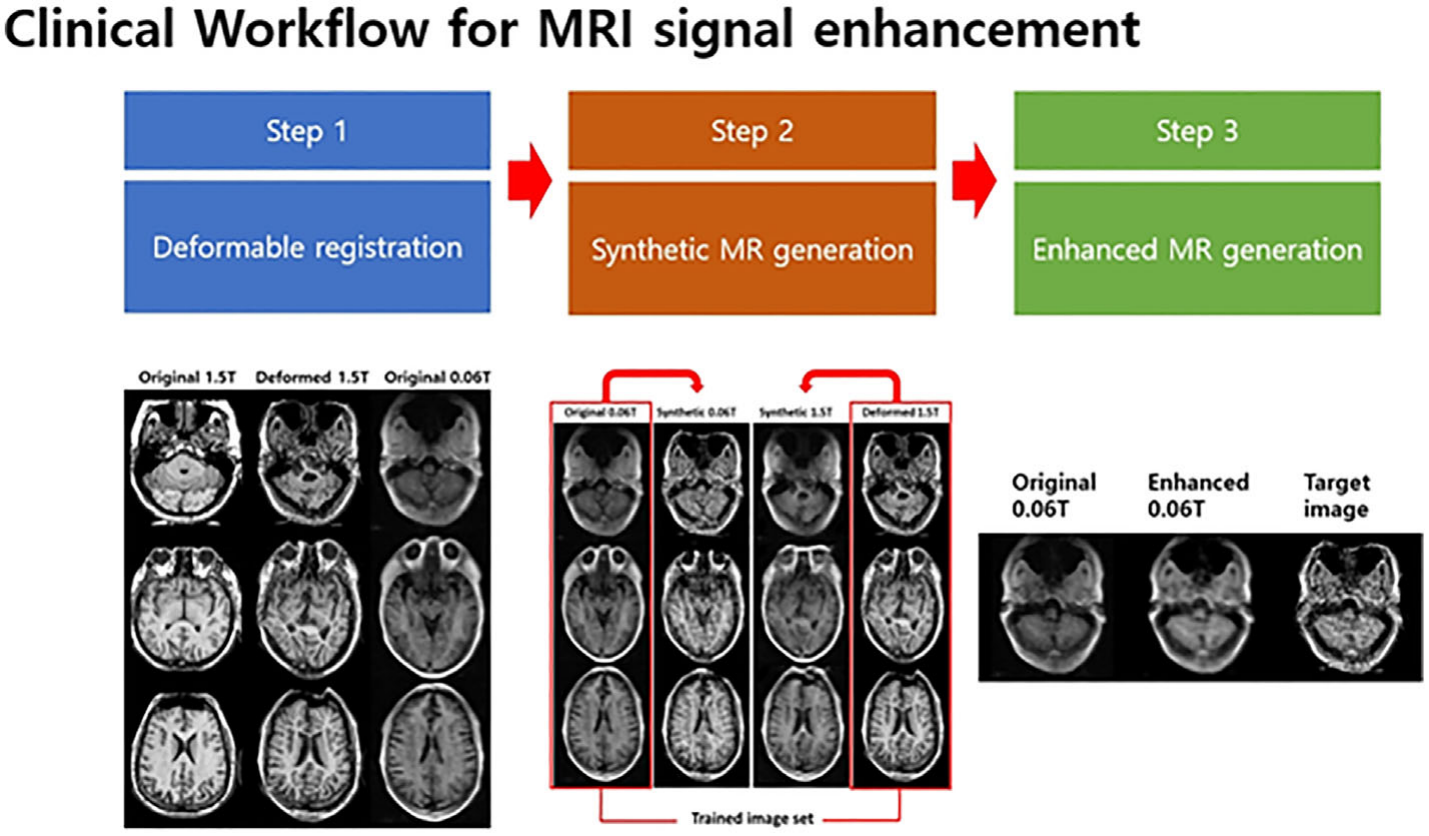
Clinical flow for enhancement of unpaired MRI set.

This study suggested a three-step clinical flow for generating enhanced MR images using 0.06 Tesla MRI, as shown in **Figure 2**. In the first step, a deformable registration process was used to match different positions and sizes of the unpaired MR image set (0.06 Tesla MR and 1.5 Tesla MR). A midpoint independent deformable registration method (29) was applied to deform a 1.5 Tesla MR image based on 0.06 Tesla MR image. In the second step, a cyclic-GAN was applied to generate a synthetic MR images from the deformed or original 1.5 Tesla MR image. This synthetic MR image was then used as a reference image in the third step to generate enhanced MRI.

To investigate the qualities of the enhanced MRI, three methods (methods 1, 2 and 3) were used with the clinical flow. In method 1, an enhanced MR image was generated from the original 0.06 Tesla MR image using the deformable registration and conventional-GAN model (without the cyclic-GAN model). We prepared training image sets with unpaired 0.06 and 1.5 Tesla MR images in each slice. Then, deformable registration was applied in the unpaired image sets. In each step, an experienced clinical physicist reviewed the process and registration results.

In method 2, the deformed 1.5 Tesla MR image (after deformable registration) and original 0.06 Tesla MR image were used as the input/reference images for the synthetic MRI generation in the cyclic-GAN. At this stage, there were two synthetic images—one was a synthetic MR image (from the original 0.06 Tesla MR image) that resembled the deformed 1.5 Tesla MR image, and the other was a synthetic MR image (from the deformed 1.5 Tesla MR image) that resembled the original 0.06 Tesla MR image. Among them, the synthetic MR image from the original 0.06 Tesla MR image was used as the reference image in the conventional-GAN model.

In method 3, the original 1.5 Tesla MR image (without deformable registration) and original 0.06 Tesla MR image were used as input/reference images for the synthetic MRI generation in the cyclic-GAN. Then, the synthetic MR image from the original 0.06 Tesla MR image (which resembled the original 1.5 Tesla MR image) was used as the reference image in the conventional-GAN model.

**Table 1** shows the clinical flow and the three methods to enhance the MR images with deformable registration, cyclic-GAN, and conventional-GAN deep learning models.

**Table 1:** Workflow with deformed MR image and without synthetic MR image for method 1, 2 and 3.

Method/Flow		Step 1	Step 2		Step 3
Method1	Model	Deformable registration	Cyclic-GAN		Conventional-GAN
	Input	Original 1.5T MR image	Not applicable		Original 0.06T MR image
	Reference	Original 0.06T MR image			Deformed 1.5T MR image
	Output	Deformed 1.5T MR image			Enhanced 0.06T MR image
Method2	Model	Deformable registration	Cyclic-GAN		Conventional-GAN
	Input	Original 1.5T MR image	Original 0.06T MR image	Deformed 1.5T MR image	Original 0.06T MR image
	Reference	Original 0.06T MR image	Deformed 1.5T MR image	Original 0.06T MR image	Synthetic MR image from 0.06T MR image with deformed 1.5T MR image
	Output	Deformed 1.5T MR image	Synthetic MR image from 0.06T MR image (used)	Synthetic MR image from deformed 1.5T MR image (not used)	Enhanced 0.06T MR image
Method3	Model	Deformable registration	Cyclic-GAN		Conventional-GAN
	Input	Not applicable	Original 0.06T MR image	Original 1.5T MR image	Original 0.06T MR image
	Reference		Original 1.5T MR image	Original 0.06T MR image	Synthetic MR image from 0.06T MR image with original 1.5T MR image
	Output		Synthetic MR image from 0.06T MR image (used)	Synthetic MR image from original 1.5T MR image (not used)	Enhanced 0.06T MR image

(0.06T and 1.5T indicate 0.06 Tesla and 1.5 Tesla respectively).

The software system for deep learning algorithm includes Python 3.7.7, TensorFlow 2.3.1, NumPy 1.18.5, OpenCV 4.4.0, Matplotlib 2.2.3, pickleshare 0.7.5, SimpleiTK 2.0.1, SciPy 1.1.0, and CUDA 11.0 with a Nvidia 12 GB Titan X GPU.

### Training and Evaluation for Conventional-GAN (Step 2) and Cyclic-GAN (Step 3)

**This study used a training set of 26 slices of T1W unpaired image set.** For the 2^nd^ step, a cyclic-GAN model (23) (https://github.com/simontomaskarlsson/GAN-MRI) was used to generate synthetic MR images using the 0.06 **Tesla** MR and 1.5 **Tesla** MR images. The training set was used for a test because the aim of this study was to determine the feasibility of using the enhanced MRI signals from the 0.06 **Tesla** MRI. The evaluation process of the algorithm is presented in (23). The training process was performed with the original 0.06 **Tesla** MR image and deformed 1.5 **Tesla** MR image (method 2) or original 1.5 **Tesla** MR image (method 3), as shown in **Table 1**. The training of neural networks is commonly supervised, that is, the training requires the corresponding ground truth for each input sample. In image-to-image translation, this implies that paired images from both the source and target domains are needed. To alleviate this constraint, a cyclic-GAN can work with unpaired training data. In the training, 180 epochs were used and six model comparisons were conducted.

**Figure 3:**
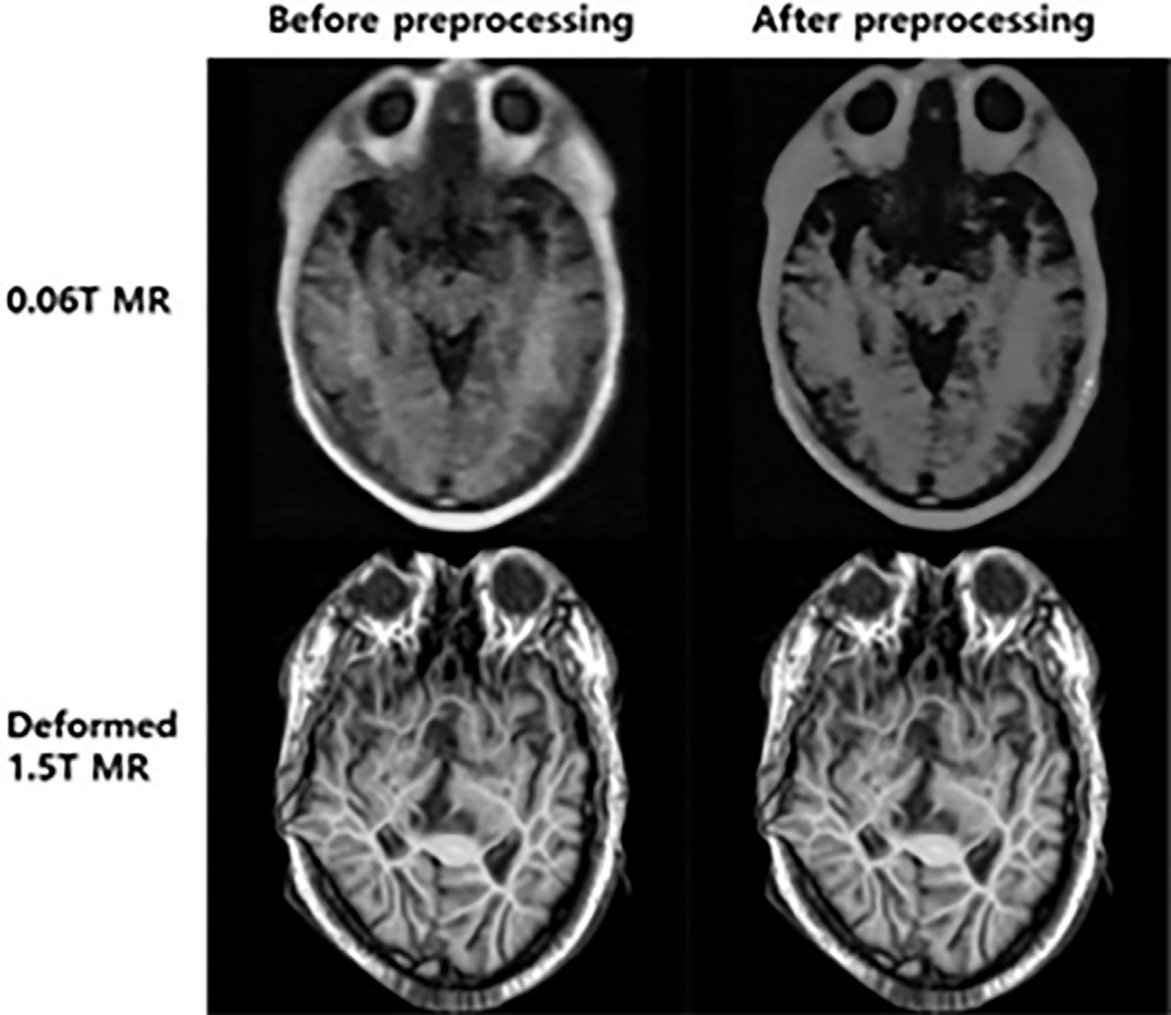
Reduced intensity after preprocessing of MR images (Han’s model).

In the 3^rd^ step, the training process was performed with the reference image of the generated synthetic MRI from the 0.06 **Tesla** MR image with the 1.5 **Tesla** MR image (deformed or original) and the input image of the original 0.06 **Tesla** MR image. Han’s model (20) was used for the conventional-GAN in the 3^rd^ step (https://github.com/ChengBinJin/MRI-to-CT-DCNN-TensorFlow). In previous work on synthetic CT generation from MR images (18, 20–22), preprocessing of the CT and MR image sets was needed. In these previous studies, after preprocessing, the input image (original CT) and reference image (original MR) were converted to resampled CT images and mark out MR images that were used for training for synthetic CT image generation. However, this study used MR images for both the input and output. If a preprocessing algorithm was used for the image set, the intensity of the 0.06 **Tesla** MR image (input image) became low, as shown in **Figure 3**. Thus, the original 0.06T MR image and synthetic MR image were used without preprocessing, even though preprocessing was performed. Mask images that were generated during this preprocessing were used for loss function training and evaluation.

In the conventional-GAN, the weights and biases of trainable filters in the convolutional layers and deconvolutional layers were trained by minimizing a loss function. The loss function was defined as the mean absolute error (MAE) between *OS_i_* and *IS_i_* within the body mask:

$$loss=\frac{1}{N}\Sigma_{i=1}^{N}|{OS}_{i}-{IS}_{i}|,$$

Where N is the number of voxels inside the body masks of the MR images, and *OS_i_* and *IS_i_* represent the intensity values of the ith voxel in the output image and input image, respectively.

In the cyclic-GAN, the goal was to learn a mapping function between the *OS* and *IS* domains given training samples. Thus, in addition to the adversarial loss (the above equation for the MAE) to both mapping functions, the learned mapping function was checked for cyclic consistency. Each image from the domain also had to satisfy the backward cycle consistency (cycle consistency loss) (24).

For a quantitative analysis of image matching between the images in step 3, a correlation factor (f) was calculated for the deformed and synthetic images as follows:

$$f=\frac{\Sigma_{m}\Sigma_{n}(A_{mn}-\bar{A})(B_{mn}-\bar{B})}{\sqrt{\left(\Sigma_{m}\Sigma_{n}{\left(A_{mn}-\bar{A}\right)}^{2})(\Sigma_{m}\Sigma_{n}{\left(B_{mn}-\bar{B}\right)}^{2}\right)}},$$

Where A and B are the two image sets being compared, $\bar{A}=mean(A)$ and $\bar{B}=mean(B)$.

To analyze the performance of the enhanced image, the enhancement ratio (S1/S2) and signal-to-noise (SNR) ratio of the output image based on the input image were investigated, where S1 and S2 were the average values of intensity for the input image (original 0.06T MR image) and output image (enhanced 0.06 **Tesla** MR image), respectively.

## Results

### Image Matching With Deformable Registration and Synthetic MR Image Generation in Cyclic-GAN (Step 1 and 2)

**Figure 4:**
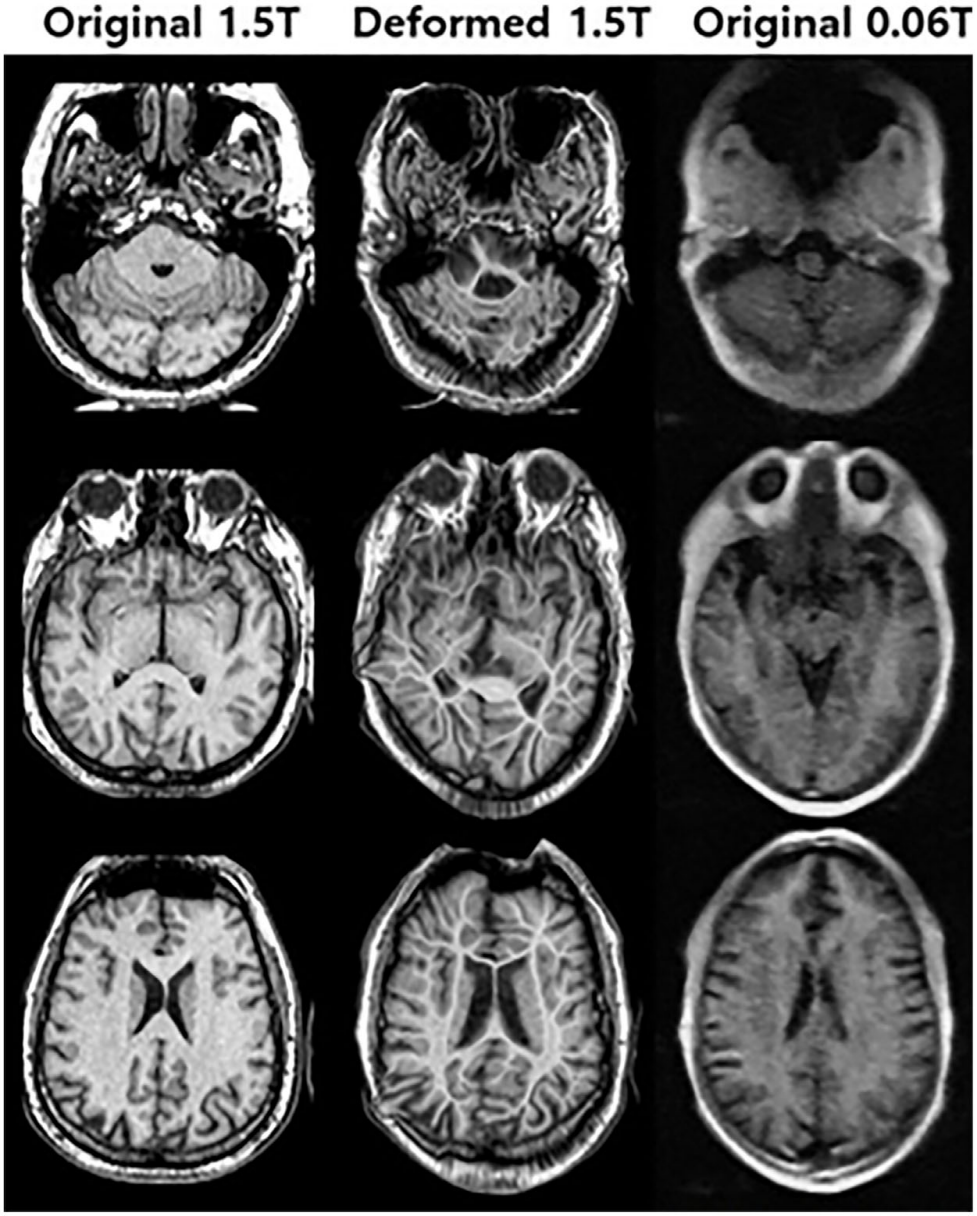
Deformed 1.5 **Tesla** MR image from original **1.5 Tesla** MR image according to 0.06 **Tesla** MR image in step 1 (deformable registration).

**Figure 4** shows an example of the deformable registration. In the 1^st^ step, midpoint independent deformable registration was applied to the original 1.5 **Tesla** MR image according to the 0.06 **Tesla** original MR image. Because unpaired sets (from different subjects) were used in this study, the initial shapes of the two original images were **quite** different. After deformable registration, the outer shape of the 1.5 **Tesla** image matched well with the original 0.06 **Tesla** image; however, the internal structure broke down compared to the original structure.

**Figure 5:**
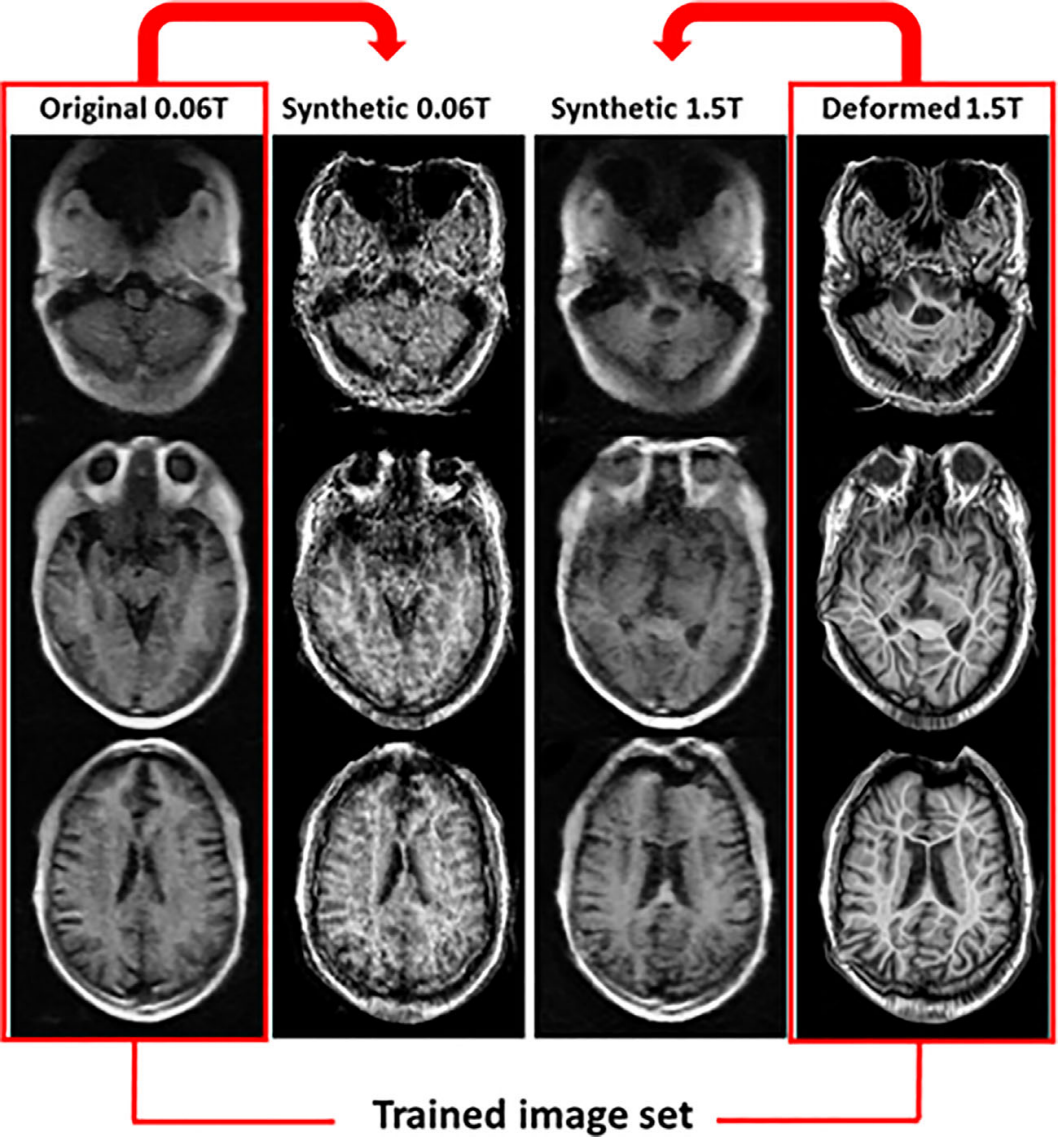
Synthetic MR images from original 0.06 **Tesla** and deformed 1.5 **Tesla** MR images in step 2 (cyclic-GAN model, method 2).

In the 2^nd^ step, two methods were used to generate the synthetic MR images. One used **deformed MR images** (method 2), and the other **used original MR images** (method 3). **Figure 5** shows the results for synthetic MR images using the original 0.06 **Tesla** MR image and deformed 1.5 **Tesla** MR image. The original 0.06 **Tesla** MR image and deformed 1.5 **Tesla** MR image were trained to resemble each other using a cyclic-GAN model. The synthetic 0.06 **Tesla** MR image and 1.5 **Tesla** MR image were generated from the original 0.06 **Tesla** and deformed 1.5 **Tesla** images, respectively. In this case, the deformed 1.5 **Tesla** images were matched with the original 0.06 **Tesla** images in relation to their sizes and positions which generated synthetic 0.06 **Tesla** images with sizes and positions similar **in** those of the original 0.06 **Tesla** images.

**Figure 6:**
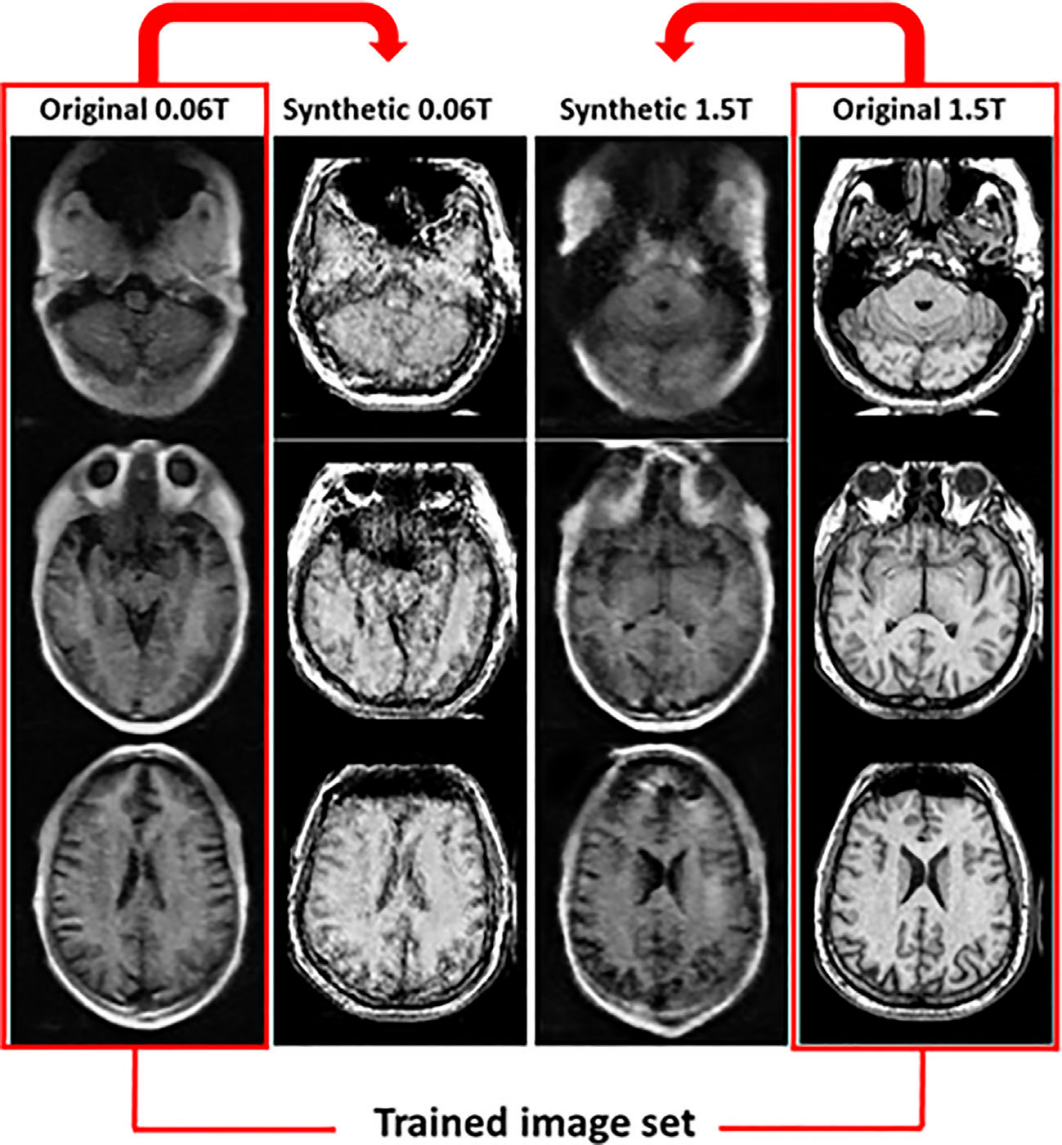
Synthetic MR images from original 0.06 **Tesla** and original 1.5 **Tesla** MR images in step 2 (cyclic-GAN model, method 3).

After deformable registration, some images showed poor quality as like artifact due to the distortion of the image. This is an effect of the deformation with an unpared image set. Thus we studied method 3. In method 3, the synthetic MR images were generated from the original 0.06 **Tesla** and original 1.5 **Tesla** MR images. In this case, the synthetic 0.06 **Tesla** images showed a higher quality for the internal structure; however, the outer shape (position and size) was close to that of the original 1.5 **Tesla**. **Figure 6** **shows the results for synthetic MR images using the original 0.06 Tesla MR image and the original 1.5 Tesla MR image**.

**Figure 7:**
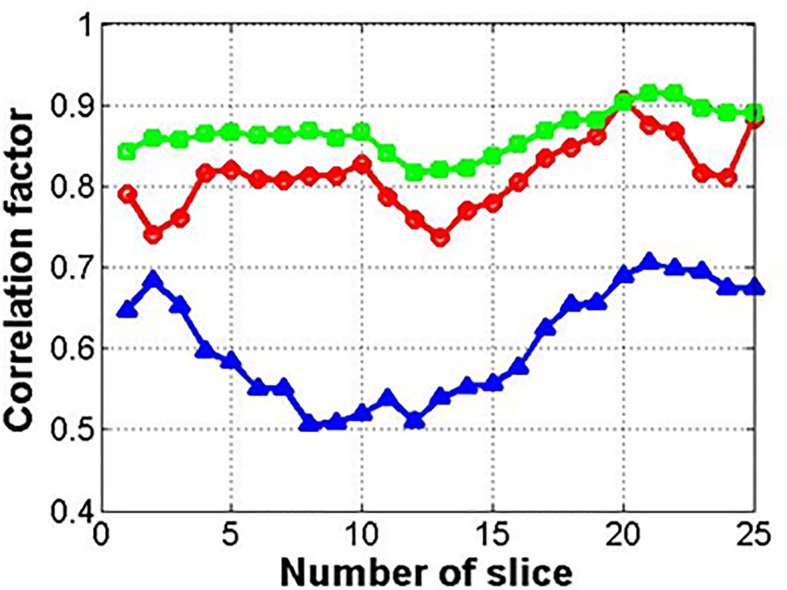
Correlation factor between input and reference images (circle, square, and triangle marks indicate deformed image, synthetic image from deformed one, and original one, respectively).

**Figure 7** shows the results for the correlation factor of the deformed images (circle) and synthetic images from the deformed (square) or original images (triangle). Among the three methods, method 2 with the related synthetic MR image had the highest correlation factor. The synthetic images from the original image (not deformed) showed the lowest correlation factor.

### Enhanced MR Generation With Conventional GAN Model (Step 3)

**Figure 8:**
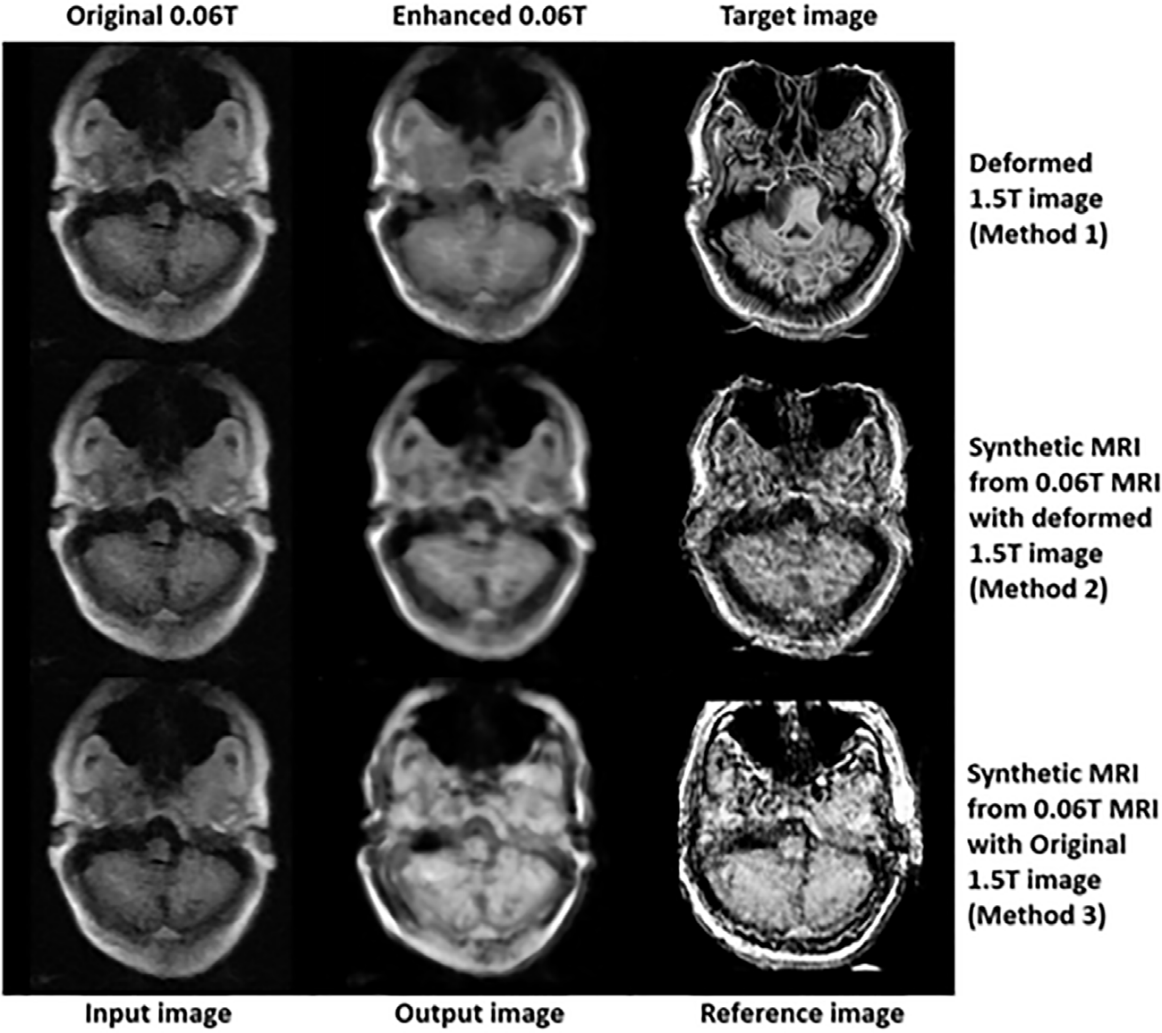
Results of enhanced MRI of slice no. 5 in step 3 (conventional-GAN model).

In the final step (3^rd^ step), deformed 1.5 **Tesla** and synthetic 0.06 **Tesla** images with the deformed 1.5 **Tesla** or original 0.06 **Tesla** MR images were used as the reference images for the generation of enhanced MR images using the conventional-GAN model. **Figures 8** **–** **10** show the results for the generated enhanced 0.06T MR image (output image) with the training set of the original 0.06 **Tesla** image (input image) and the various target images (reference images).

**Figure 9:**
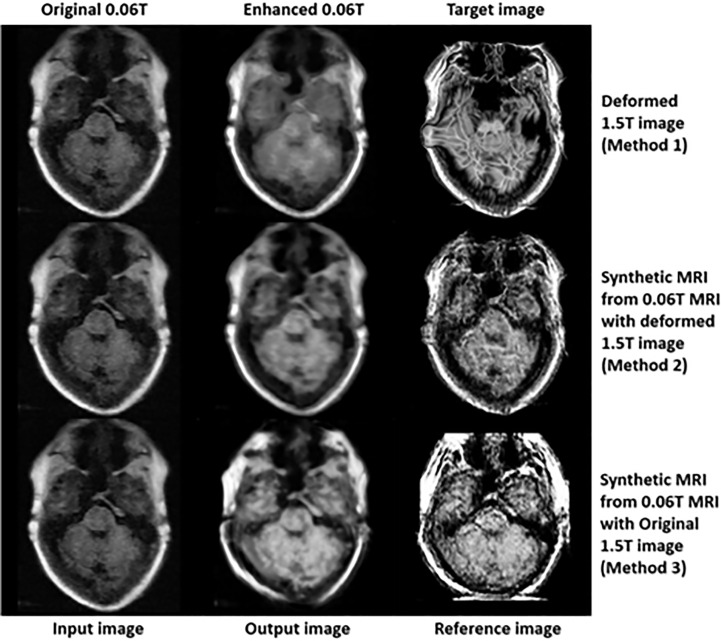
Results of enhanced MRI of slice no. 8 in step 3 (conventional-GAN model).

**Figure 10:**
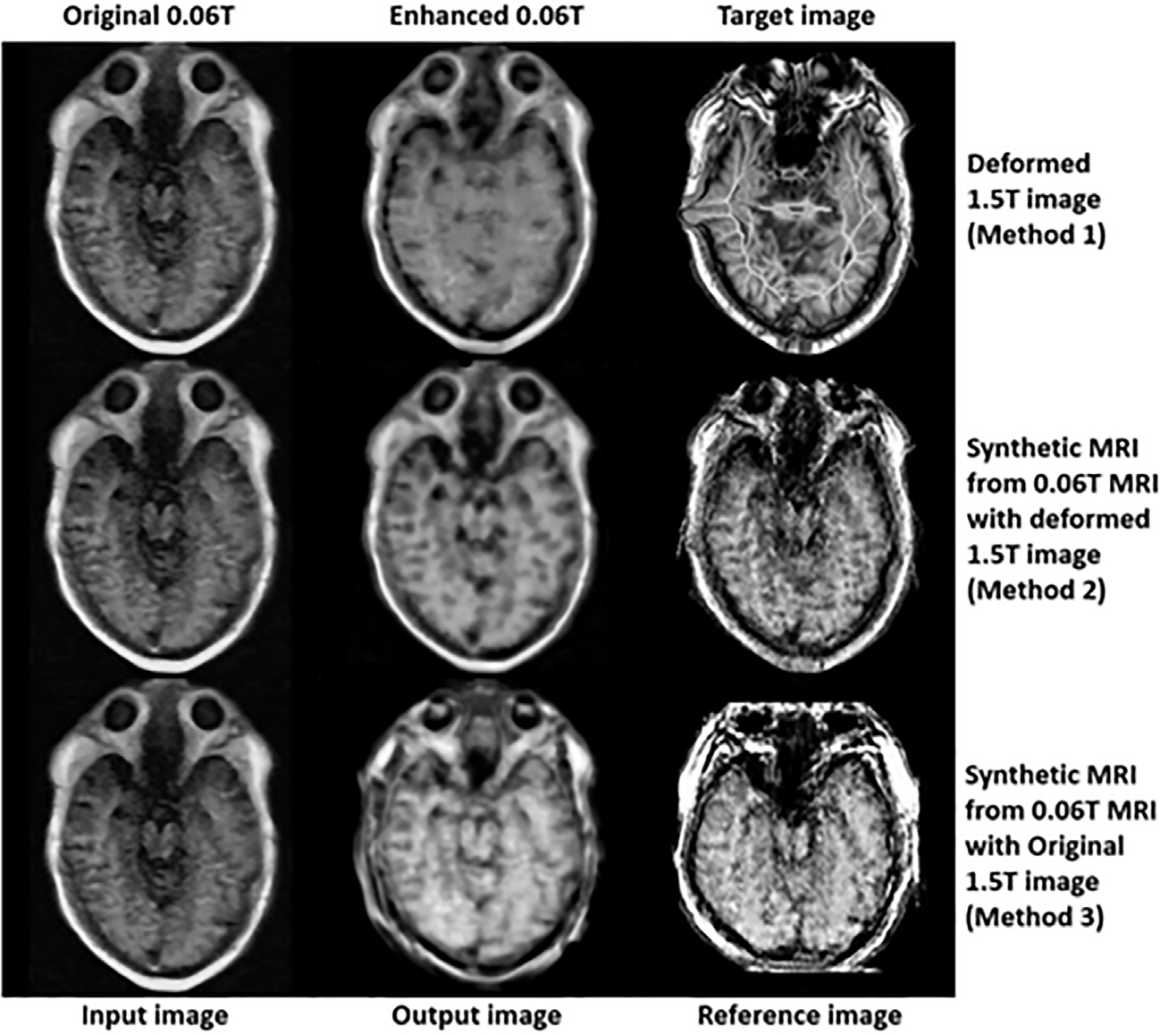
Results of enhanced MRI of slice no. 10 in step 3 (conventional-GAN model).

In **Figure 8**, the intensity of the nasopharyngeal fossa is enhanced compared to the original 0.06 **Tesla** MR image. In addition, the overall intensities of the tissue and bone are increased in the enhanced 0.06 **Tesla** MR image. The analytic structure of the nasopharyngeal fossa is seen more clearly in the trained image set of the deformed 1.5 **Tesla** image; however the overall intensity of the tissue is increased in the synthetic MR image cases.

**Figure 9** shows the signal enhancement for the nasal septum and inferior nasal concha. In addition, the signal intensities were increased in the temporal lobe, medulla oblongata and cerebellar hemisphere. The analytic structure of the nasal septum was seen more clearly after using methods 2 and 3. After method 1, there was a vague pattern in the boundary between the temporal lobe and cerebellar hemisphere, including the medulla oblongata. The shape of the enhanced 0.06 **Tesla** MR image in method 2 was well-matched to the original 0.06 **Tesla** MR image. Thus, the enhanced nasal septum revealed the anatomic structure reasonably. However, in method 3, the shape was distorted because of the different shape of the original 1.5 **Tesla** MR image in comparison to the original 0.06 **Tesla** MR image.

**Figure 10** shows the enhanced 0.06 **Tesla** MR image, especially the nasal septum, olfactory nerve, and eye lens. After using method 1, the boundary of the brainstem was unclear. The anatomical structure of the nasal septum could be seen more clearly after method 2 than method 1. In the case of method 3, the enhanced nasal septum structure seemed abnormal compared to the result from method 2. In addition, the contrast of the overall tissue area was more palpable in method 2 than in method 1.

**Figure 11:**
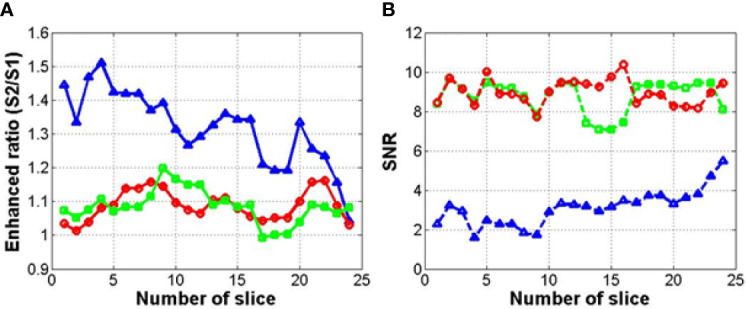
Enhancement ratio **(A)** and signal-to-noise ratio (SNR) **(B)** of enhanced MR image for each method (circle, square, and triangle marks indicate methods 1, 2, and 3, respectively).

**Figure 11** shows the results of a quantitative analysis of the enhancement of the MR images using each method. This quantitative analysis was conducted to determine the enhancement of the MR image intensity in the overall region (256×256 image size). The depth of the MR image in this study was 255 (8 bit-PNG image file). **Figure 11** shows the signal ratio of the enhanced 0.06 **Tesla** MR image to the original 0.06 **Tesla** MR image in step 3 for each method (1, 2, and 3). In **Figure 10** (A), the enhancement ratios show the ratio of the signal intensities of the enhanced MR image (S2) to that of the original MR image (S1) for method 1 (circle), method 2 (square), and method 3 (triangle). The enhancement ratios for methods 1 and 2 could increase up to 20% among slices, and the enhancement ratio for method 3 increased to approximately 50% compared to the original 0.06 **Tesla** MR image. **Figure 10** (B) shows the SNR of the enhanced 0.06 **Tesla** MR image based on the background of the original 0.06 **Tesla** image. The SNR values for methods 1 and 2 were higher than that of method 3.

**Figure 12:**
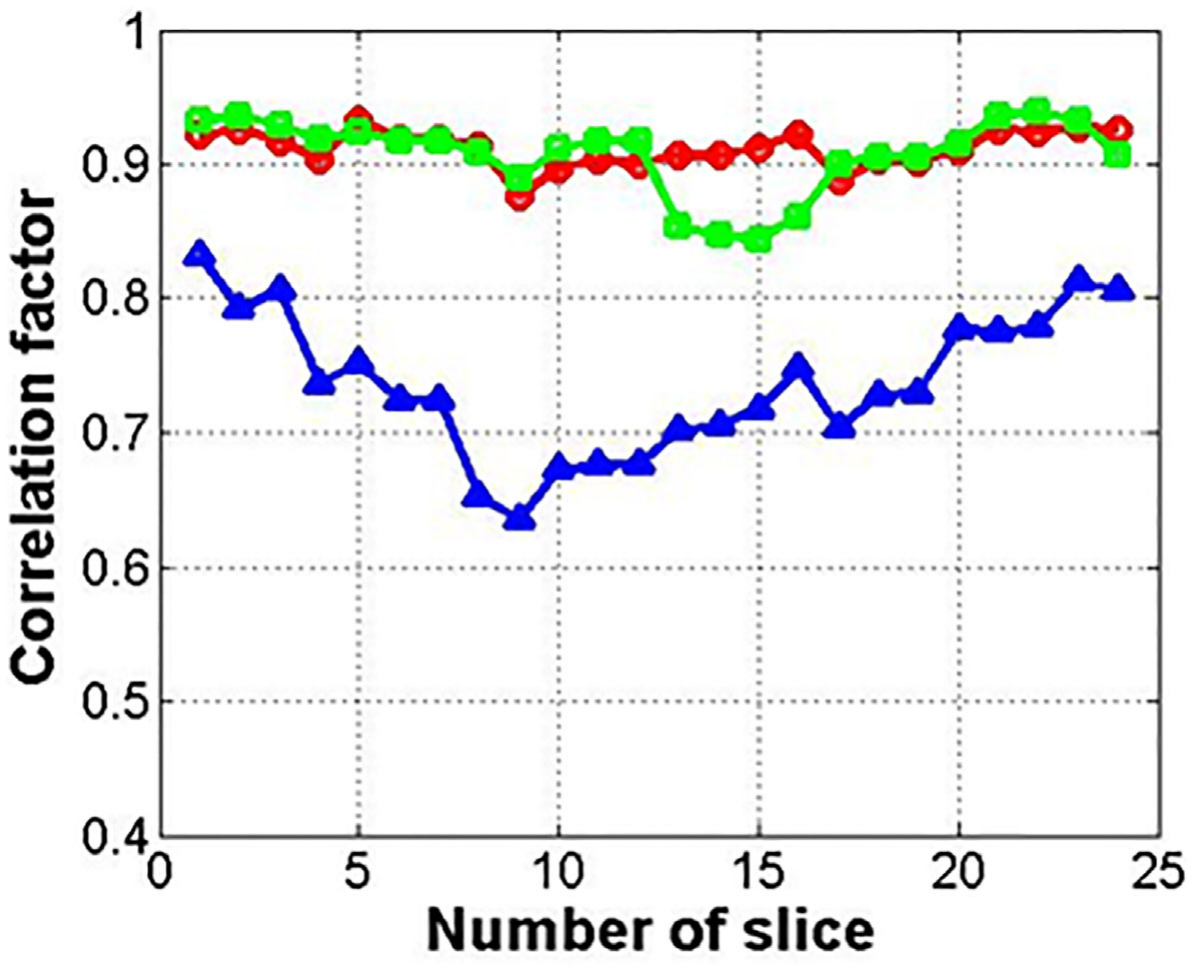
Correlation factor between input and output images for GAN (circle, square, and triangle marks indicate methods 1, 2, and 3, respectively).

**Figure 12** shows the correlation factor between the input image (original 0.06 **Tesla** MR image) and output image (enhanced 0.06 **Tesla** MR image). The circle, square, and triangle marks indicate methods 1, 2, and 3, respectively. The correlations for methods 1 and 2 are higher than that for method 3. And moreover, the correlation for the enhanced image was improved compared to the reference cases in **Figure 7**.

## Discussion

In (23), several different GAN models were investigated in a literature study (24, 25, 30–33). And cyclic-GAN (24) and UNIT (25) were selected. The generation of synthetic images took 0.0176 and 0.0478 ms per image for the cyclic-GAN and UNIT, respectively using an Nvidia 12 GB Titan X GPU with 180 epochs. In the present study, to reduce the process time, a cyclic-GAN was selected, and the generation time was 0.0183 ms per image using the same system.

Synthetic MR images are shown in **Figures 5** and **6**. In the cyclic-GAN model, the adversarial loss had to be established for both image domains (input and reference in **Table 1**) with cyclic loss consistency (24). The synthetic MR images generated from the 0.06 **Tesla** and 1.5 **Tesla** images became close to each other. In methods 2 and 3, only the synthetic MR images from the 0.06 **Tesla** MR image which resembled the 1.5 **Tesla** MR image, were used as reference images in step 3 (conventional-GAN). The synthetic MR images from the 1.5 **Tesla** MR image which was similar **in** the 0.06 **Tesla** MR image, were not used in this study.

**Table 2:** Evaluation factors of the enhanced MR images for three method.

Method/Item	Correlation Factor	Enhancement Ratio	Signal-to-Noise Ratio (SNR)
Method1	0.91±0.01	1.09±0.04	8.99±0.65
Method2	0.91±0.03	1.09±0.05	8.76±0.83
Method3	0.74±0.05	1.32±0.11	3.10±0.89

In the case of method 1, a deformable registration process was applied. However, although this process made the shape (size and position) of the original 1.5 **Tesla** MR image close to that of the original 0.06 **Tesla** MR image, the internal anatomical structure broke down, as shown in **Figure 4**. This was due to the use of unpaired image sets between the original 0.06 **Tesla** and 1.5 **Tesla** MR images. Then, enhanced MR images were generated in step 3 through the conventional-GAN model without step 2 (synthetic MR image generation with cyclic-GAN). The shape of the enhanced MR image was close to the original 0.06 **Tesla** image. However, the internal structure was not as clear compared to the other methods (methods 2 and 3), as shown in **Figures 9** and **10**.

In the case of method 2, the synthetic MR image generated with the deformed 1.5 **Tesla** MR image showed a higher performance in terms of the shape (size and position) and internal structure than method 1. The nasal structure and border of the brainstem were seen more clearly than with method 1 (**Figures 8–** **10**). However, the enhancement ratio was similar to the pattern over all the slides (**Figure 11**).

To avoid the distortion of the deformed image, method 3 used the original 1.5 **Tesla** MR image reference image in step 3. The nasal structure and eye lens were seen the most clearly, and the enhancement ratio showed the highest value over all the slides. However, method 3 showed poor performance for the shape, although it showed a relatively higher quality for the internal structure (**Figures 10**, **11**). This could result in another distortion of the enhanced MR images, which was the limitation of using the unpaired image set.

The correlation factor indicates the resemblance between the two images—the input image and output image. In **Figure 7**, which shows the results after cyclic-GAN, the input and output images were the original 0.06 Tesla MR image and the deformed or synthetic images according to methods 1, 2, and 3. In **Figure 12**, the calculated correlation factors between the enhanced MR images and the original images are slightly higher than the results in **Figure 7**. However, the difference was not very significant. For the enhancement ratio, a significant difference between the input and output images was generated after the conventional-GAN, as shown in **Figure 11**. This implied that deformable registration and synthetic MR image generation with the cyclic-GAN had an important role in the correlation in the workflow. Also, conventional-GAN was contributed to the signal enhancement of the MR images.

For the quantitative analysis, we calculated the correlation factor, enhancement ratio, and signal-to-noise ratio as the evaluation factors for the image quality of the enhanced MR image. These factors were compared among the three methods as shown in **Table 2**.

Methods 1 and 2 show almost similar evaluation factors over all items with a high correlation factor and about 10% enhancement of the MR image signal. In the case of method 3, the enhancement ratio was higher than those of methods 1 and 2 (over 30%), however, the correlation factor decreased significantly. Though the signal enhancement was superior, the image correlation between the input and output images is more important in clinical application. Also, while the evaluation factors showed similar values in methods 1 and 2, the qualitative analysis showed that method 2 was the best option in this study due to its clear internal structure as compared to method 1.

In our study, we used 26 images of the unpaired image sets (26 0.06 Tesla MR images and 26 1.5 Tesla MR images). These images were used in training and evaluation. Also, the same images were used in testing for cyclic-GAN and conventional-GAN models. Thus, we do not have enough data for presenting statistical information in this study. To overcome the current limitation of our study, more data should be mined and studied further.

## Conclusions

This study investigated a deep learning-based three-step workflow to increase the signal intensity for MR images and determine feasibility of generating an enhanced MR image with an unpaired image set. Deformable registration is one of the options to create a reference image from the unpaired image set in the GAN model. Synthetic images using a cyclic-GAN were suitable for the creation of a reference image of the unpaired set in the conventional-GAN model. To avoid the image distortion of the final enhanced MR image, using the synthetic deformed MR image was found to be better than using the original synthetic MR image in the cyclic-GAN. The conventional-GAN and cyclic-GAN models could be used to efficiently generate enhanced MR images in low-magnetic-field (0.06 **Tesla**) MRI. To obtain the maximum efficiency, the optimized workflow (method 2 in this study) could be used for clinical purposes.

## Data Availability Statement

The raw data supporting the conclusions of this article will be made available by the authors, without undue reservation.

## Author Contributions

DY, YC, CR and EL conceived of the presented idea. DY and BM developed the theory and performed the computations. JC and EK verified the analytical methods. DY, YC, CR and EL encouraged EK to investigate medical efficiency and supervised the findings of this work. All authors discussed the results and contributed to the final manuscript. All authors contributed to the article and approved the submitted version.

## Funding

This work was supported by the grant of Research Institute of Medical Science, Catholic University of Daegu (2020).

## Conflict of Interest

The authors declare that the research was conducted in the absence of any commercial or financial relationships that could be construed as a potential conflict of interest.
